# Cytostéatonécrose néonatale compliquée d’hypercalcémie majeure

**DOI:** 10.11604/pamj.2018.29.86.14234

**Published:** 2018-01-30

**Authors:** Assiya El Kettani, Nabiha Kamal

**Affiliations:** 1 Laboratoire de Biochimie, CHU Ibn Rochd, Université Hassan II, Casablanca, Maroc

**Keywords:** Cytosteatonécrose, nouveau-né, hypercalcémie, Cytosteatonecrosis, infant, hypercalcaemia

## Abstract

We here report the case of a female infant with perinatal asphyxia requiring resuscitation. She was referred with sclerema neonatorum which had been observed by parents on her tenth day of life. The lesions appeared as hard erythematous nodular plaques, suggesting neonatal cytosteatonecrosis in the gluteal region. Patient’s evolution was marked by progressive hypercalcemia reaching a peak of 128 mg/L (80-110 mg/L) for which re-hospitalization was required 1 month after birth. Renal ultrasound showed medullary nephrocalcinosis. Treatment was based on hyperhydration associated with diuretics, corticosteroids and abstention from vitamin D administration as well as on clinical and laboratory monitoring of serum calcium levels. A month later, cutaneous lesions were regressing and calcium levels were normalizing. The patients is still undergoing ultrasound monitoring.

## Image en médecine

Nouveau-né de sexe féminin, ayant présenté une asphyxie périnatale pour laquelle il a été réanimé. Il a été référé ensuite pour sclérème cutané, constaté par les parents au dixième jour de vie. Les lésions étaient des placards érythémateux nodulaires indurés, évoquant une cytostéatonécrose néonatale au niveau des régions fessières. L’évolution a été notée par une hypercalcémie progressive atteignant 128 mg/L (80-110 mg/L), ce qui a indiqué une ré-hospitalisation à un mois de vie. L’échographie rénale a objectivé une néphrocalcinose médullaire. Le traitement a associé une hyperhydratation, des diurétiques, des corticoïdes et une abstention d’administration de la vitamine D avec une surveillance clinique et biologique de la calcémie. L’évolution a été vers la régression des lésions cutanées et la normalisation de la calcémie un mois après. La surveillance échographique de la néphrocalcinose est toujours en cours.

**Figure 1:**
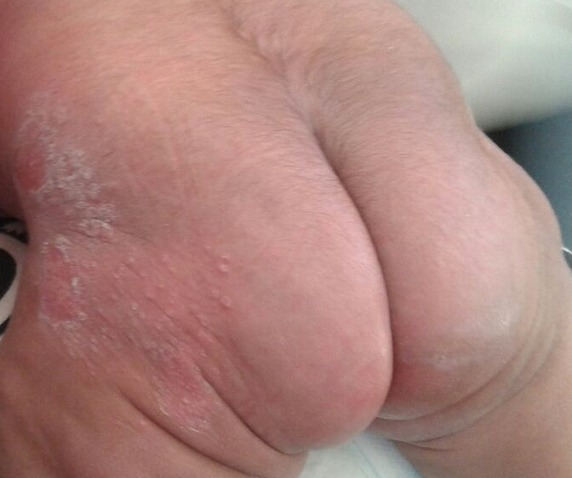
Placard érythémateux nodulaire induré des fesses évoquant une cytostéatonécrose

